# A novel enediyne‐integrated antibody–drug conjugate shows promising antitumor efficacy against CD30^+^ lymphomas

**DOI:** 10.1002/1878-0261.12166

**Published:** 2018-01-26

**Authors:** Rong Wang, Liang Li, Shenghua Zhang, Yi Li, Xiaofei Wang, Qingfang Miao, Yongsu Zhen

**Affiliations:** ^1^ Institute of Medicinal Biotechnology Chinese Academy of Medical Sciences Peking Union Medical College Beijing China

**Keywords:** ADC, CD30‐targeting, cytotoxicity, lymphoma, therapeutic efficacy

## Abstract

CD30 is a 120‐kDa type I transmembrane glycoprotein belonging to the tumor necrosis factor receptor superfamily. Overexpression of CD30 has been reported in Hodgkin's lymphoma (HL) and anaplastic large‐cell lymphoma (ALCL). CD30‐targeted treatment with antibody–drug conjugates (ADCs) can lead to promising clinical benefit. Lidamycin (LDM), consisting of an apoprotein LDP and an active enediyne chromophore AE, is a member of the enediyne antibiotic family and one of the most potent antitumor agents. AE and LDP can be dissociated and reconstituted under certain conditions *in vitro*. LDM is an ideal payload for the preparation of ADCs. In this study, we show the generation, production, and antitumor activity of anti‐CD30‐LDM, a novel ADC which consists of the intact anti‐CD30 antibody and LDM. First, the anti‐CD30‐LDP fusion protein was constructed and expressed in CHO/dhFr^−^ cells. Anti‐CD30‐LDP showed specific and high‐affinity binding to CD30 and could be internalized into target cells. It also exhibited excellent tumor‐targeting capability *in vivo*. Next, anti‐CD30‐LDM was prepared by assembling the enediyne molecule AE to the fusion protein anti‐CD30‐LDP. Anti‐CD30‐LDM was highly cytotoxic to HL and ALCL cell lines, with IC_50_ values of 5–50 pm. It can also induce cell apoptosis and G2/M cell cycle arrest. In the Karpas299 xenograft model, the tumor growth was inhibited by 87.76% in mice treated with anti‐CD30‐LDM and with no discernible adverse effects. Taken together, anti‐CD30‐LDM shows attractive tumor‐targeting capability and antitumor efficacy both *in vitro* and *in vivo* and could be a promising candidate for the treatment of CD30^+^ lymphomas.

**Abbreviations**
ADC: antibody–drug conjugate
ADCC: antibody‐dependent cellular cytotoxicity
ALCL: anaplastic large‐cell lymphoma
BV: brentuximab vedotin
CHO: Chinese hamster ovary
FACS: fluorescence‐activated cell sorting
HL: Hodgkin's lymphoma
MFI: mean fluorescence intensity
MMAE: monomethyl auristatin E
NOD/SCID: nonobese diabetic/severe combined immune deficiency
PARP: poly (ADP‐ribose) polymerase
PBMCs: peripheral blood mononuclear cells

## 1 Introduction

Hodgkin's lymphoma (HL) is a relatively uncommon human malignancy with estimated 8260 newly diagnosed cases in the United States in 2017 (Siegel *et al*., 2017). Anaplastic large‐cell lymphoma (ALCL) is an aggressive form of malignant neoplasm of T‐cell lymphoma that accounts for approximately 10–15% of pediatric/adolescent non‐Hodgkin's lymphomas (NHLs) and only 2% of adult NHLs (Boi *et al*., 2015). With the advances made in frontline combinational chemotherapy and radiotherapy, the cure rate of HL and ALCL has been significantly increased. Nevertheless, a small percentage of patients are either resistant to the first‐line therapy or relapse after initial treatment (Turner *et al*., 2016; Younes and Ansell, 2016). There are still approximately 50% of relapsed/refractory patients after autologous hematopoietic stem cell transplantation (ASCT) (Angelopoulou *et al*., 2017). Thus, it is essential to develop new and better treatments for HL and ALCL.

The treatment paradigms of malignant cancers have been changed following the advent of monoclonal antibody–drug conjugates (ADCs), by which both Hodgkin's and non‐Hodgkin's lymphoma profited largely (Jagadeesh and Smith, 2016). In ADCs, cytotoxic drugs are linked to antibodies that recognize cancer cell antigens, and thus, the cytotoxic drugs were only delivered to the cells of interest (Polson *et al*., 2007). So far the most successful ADCs are brentuximab vedotin (BV) and trastuzumab‐DM1 (Lewis Phillips *et al*., 2008; Senter and Sievers, 2012), and they reinforce the use of ADCs as a promising treatment modality for use in oncology.

CD30, a 120‐kDa type I transmembrane glycoprotein belonging to the tumor necrosis factor receptor superfamily (Vaklavas and Forero‐Torres, 2012), is abundantly expressed in HL, ALCL, and germ cell tumor (Muta and Podack, 2013). Normally, CD30 expression is limited to activated B and T lymphocytes and restricted on normal tissues (Deutsch *et al*., 2011). Thus, CD30 is a prominent and validated target for antibody‐based therapies due to the high and selective expression by malignant cells (Hombach *et al*., 2016). One of the most successful cases is BV, an anti‐CD30 antibody conjugated to the microtubule‐disrupting agent monomethyl auristatin E (MMAE) by a protease‐cleavable linker (Chen *et al*., 2016). The U.S. Food and Drug Administration (FDA) approved BV for patients with relapsed HL and relapsed systemic ALCL in August 2011 (de Claro *et al*., 2012; Deng *et al*., 2013). BV has changed the therapeutic scenario of relapsed or refractory HL and ALCL with the safety and efficacy in a series of clinical studies (Fanale *et al*., 2012; Gopal *et al*., 2015; Pro *et al*., 2012; Younes *et al*., 2010, 2012). Briefly, targeting CD30 is an effective treatment approach for HL and ALCL.

Lidamycin (LDM, original named C‐1027), an antitumor antibiotic, shows extremely potent cytotoxicity toward human cancer cells with IC_50_ values 1000‐fold lower than that of Adriamycin *in vitro* and exhibits remarkable inhibition on a panel of transplantable tumors in mice (Shao and Zhen, 2008). It contains an active enediyne chromophore (AE) responsible for the extremely potent bioactivity and a noncovalently bound apoprotein LDP, which forms a hydrophobic pocket to protect the chromophore (Guo *et al*., 2010). Notably, AE and LDP can be dissociated and reconstituted under certain conditions *in vitro* (Tanaka *et al*., 2001). LDM can induce cell damage including apoptosis, cell cycle arrest, and DNA double‐strand breaks (Kennedy and Beerman, 2006). In a word, LDM is deemed to be a desirable cytotoxic payload to antibody‐targeted therapeutics due to its extremely potent cytotoxicity to cancer cells.

In this study, we generated a novel ADC, anti‐CD30‐LDM, which composed of a chimeric anti‐CD30 monoclonal antibody (mAb), nonprotease peptide linkers, and LDM. Then, we demonstrated its preclinical characterization as well as potential for the treatment of CD30^+^ lymphomas.

## 2 Materials and methods

### 2.1 Cell lines

The CD30‐positive Hodgkin‐derived (HD) cell lines L540 and L428 were obtained from Creative Bioarray, Inc. (Shirley, NY, USA), and the ALCL lines Karpas299 and SU‐DHL‐1 were purchased from BIOPIKE (Beijing, China), whose head office was in United States. The Burkitt lymphoma cell lines Raji and Daudi were provided by our laboratory, and the acute promyelocytic leukemia cell line HL60 was obtained from the Cell Center of Peking Union Medical College (Beijing). The CHO/dhFr^−^ cell line was purchased from the American Type Culture Collection (ATCC, Manassas, VA, USA). L540 and HL60 cell lines were cultured in RPMI‐1640 supplemented with 20% fetal bovine serum (FBS), while L428, Karpas299, SU‐DHL‐1, Raji, and Daudi cell lines were cultured in RPMI‐1640 in 10% FBS. CHO/dhFr^−^ cell line was cultured in Iscove's modified Dulbecco's medium supplemented with 0.1 mm hypoxanthine, 0.016 mm thymidine, 0.002 mm methotrexate hydrate, and 10% FBS. All cell lines were cultured in a humidified incubator, maintained at 37 °C with 5% CO_2_.

### 2.2 Construction and expression of anti‐CD30 antibody fusion proteins

The DNA sequences of variable regions of heavy chains (VHs) and light chains (VLs) of the chimeric anti‐CD30 antibody cAC10 were referred to US patent publication (Pub. No. US2008/0267976 A1). We designed the LDP sequence of LDM to the N‐terminal of VL with a noncleavable peptide SGGPEGGS. All the DNA fragments were synthesized by GenScript Company (Nanjing, China). And the expression vector pIZDHL was kindly provided by Xiao‐yun Liu (Liu *et al*., 2011), and it contains two cytomegalovirus (CMV) promoters which were stronger than SV40 and LTR promoters. In addition, the *Bleo* and *Dhfr* genes were employed as selection markers.

For construction of anti‐CD30‐LDP, DNA fragments encoding the VH and LDP‐SGGPEGGS‐VL protein sequences were, respectively, cloned into the expression vector pIZDHL which carried the gene sequence encoding the human IgG_1_ constant region, designated as pIZDHL‐anti‐CD30‐LDP. And as the control, *VH* and *VL* sequences were similarly joined to pIZDHL for the expression of chimeric anti‐CD30 antibody, designated as pIZDHL‐anti‐CD30.

For the generation of anti‐CD30‐LDP and anti‐CD30 antibody‐expressing cell lines, pIZDHL‐anti‐CD30‐LDP and pIZDHL‐anti‐CD30 were linearized and transfected into CHO/dhFr‐ cells by lipofectin transfection (Invitrogen, Carlsbad, CA, USA), respectively. Then, the cells were allowed to recover in complete medium (IMDM containing 10% FBS, 0.1 mm hypoxanthine, 0.016 mm thymidine, and 0.002 mm methotrexate hydrate) for 24 h, after which the medium was replaced with selective medium (IMDM containing 10% dialyzed FBS and 200 μg·mL^−1^ bleomycin) without hypoxanthine and thymidine. Only those cells incorporated the plasmid DNA, which carried the dihydrofolate reductase gene and bleomycin resistance gene, were able to grow in selective medium and screened by ELISA for the expression levels of indicated recombinant protein. The clones producing the highest levels of proteins were selected and cultured subsequently.

### 2.3 Purification and purity analysis of antibody‐based fusion proteins

The selected cell lines were processed by amplification culture, and then, the culture medium was changed to CHO serum‐free medium (CD OptiCHO™ Medium; Gibco, Grand Island, NY, USA) with GlutaMAX™ supplement (Gibco). The cell culture medium was collected after 10 days to purify the proteins of interest. The recombinant proteins anti‐CD30‐LDP and anti‐CD30 antibody were purified by protein G columns (HitrapTM Protein G HP; GE Healthcare, Chicago, IL, USA) according to the manufacturer's instructions, and the purification of recombinant proteins was performed with the binding buffer at pH 7.4 and the elution buffer at pH 2.5. Then, the concentrations of proteins of interest were assayed by the BCA method (Pierce BCA protein Assay Kit, Thermo Fisher Scientific, Waltham, MA, USA) with the bovine gamma globulin (BGG) standard. The purified proteins were then investigated by nonreducing and reducing SDS/PAGE gels, and the purity values were determined by HPLC.

### 2.4 Binding activity of the antibody fusion proteins *in vitro*

#### 2.4.1 ELISA

The high‐binding 96‐well plates were coated with the recombinant CD30‐Fc fusion protein (R&D Systems, Minneapolis, MN, USA). After blocking the wells with 2% BSA/PBS/0.05% Tween‐20 solution, anti‐CD30‐LDP and the control anti‐CD30 antibody were incubated at varying concentrations at 37 °C for 1 h. Then, the wells were washed with PBS/0.05% Tween‐20 (PBST) and incubated with an alkaline phosphatase goat anti‐human IgG (Fab‐specific) probe at 37 °C. The excess probe was washed from the wells with PBST, and the plate was incubated at room temperature for an appropriate time (usually 5–10 min) after adding the *p*‐nitrophenyl phosphate (*p*‐NPP) substrate solution. The optical density at 405 nm was determined using a microplate reader (Thermo Fisher Scientific).

#### 2.4.2 Surface plasmon resonance (SPR) study

The Biacore T200 SPR instrument (GE Healthcare) was used to examine the SPR reaction of antigen CD30 with anti‐CD30‐LDP or the control anti‐CD30 antibody. The CM5 sensor chip was preimmobilized with goat anti‐human antibodies. Anti‐CD30‐LDP or the control antibody was conjugated to the chip with a concentration of 1 μg·mL^−1^, and the response units (RUs) were 250 and 200, respectively. The coupling buffer for anti‐CD30‐LDP and anti‐CD30 antibody was 10 mm NaAc at pH 5.0. Various concentrations of antigen CD30 in HEPES buffer were injected at a flow rate of 30 μL·min^−1^. After each detection cycle, the sensor surface was regenerated with 3 m MgCl_2_ (flow rate: 30 μL·min^−1^, contact time: 60 s), allowing resonance signals to return to baseline values. The biacore t200 evaluation software (GE Healthcare) was used for data processing and analysis.

#### 2.4.3 Flow cytometry analysis

For antibody saturation binding, 3 × 10^5^ cells were incubated with increasing concentrations (0.001, 0.003, 0.01, 0.03, 0.1, 0.3, 1, 3 μg·mL^−1^) of anti‐CD30‐LDP diluted in ice‐cold 2% FBS/PBS (staining medium) for 1 h at 4 °C, then washed twice with ice‐cold staining medium to remove free mAbs, and incubated with 1 : 100 FITC‐conjugated goat anti‐human IgG for 1 h at 4 °C. The labeled cells were washed and resuspended in PBS, and then, the cell‐associated fluorescence was determined by FACS Calibur (BD Biosciences, San Jose, CA, USA).

### 2.5 Antibody‐dependent cellular cytotoxicity (ADCC) assays

Antibody‐dependent cellular cytotoxicity was analyzed using the Cytotoxicity Lactose Dehydrogenase Assay Kit (Dojindo, Kumamoto, Japan) according to the manufacturer's protocol. Briefly, peripheral blood mononuclear cells (PBMCs) were prepared from a single healthy blood donor by Ficoll‐Paque Plus (GE Healthcare). Cancer cells (target cells) and PBMCs (effector cells) were coincubated at an effector to target ratio of 40 : 1 in RPMI 1640 medium with 2% FBS in a 96‐well U‐bottomed plate. Anti‐CD30 antibody, anti‐CD30‐LDP, or positive control (Cetuximab; Merck KGaA, Darmstadt, Germany) was added, respectively, to each well at 10 μg·mL^−1^ and incubated for 6 h at 37 °C. The absorbance was measured at 490 nm using a microplate reader. All assays were conducted in triplicate.

### 2.6 Internalization of the antibody fusion proteins

To detect surface‐bound antibodies, the cell lines Karpas299, L540, and Raji (2 × 10^5 ^cells·mL^−1^) were incubated with 5 μg·mL^−1^ anti‐CD30‐LDP for 30 min on ice and rinsed with ice‐cold PBS; then, the cells were centrifuged to the glass slides using the cytospin and fixed with 4% paraformaldehyde for 10 min and permeabilized with 0.2% Triton X‐100 in PBS for 5 min at room temperature. After blocking the nonspecific sites with 5% normal goat serum (Jackson ImmunoResearch, West Grove, PA, USA), cells were incubated with 3 μg·mL^−1^ Alexa Fluor 488‐conjugated F(ab′)_2_ fragment goat anti‐human IgG (Jackson ImmunoResearch) for 30 min at 37 °C and then washed with PBST. The slides were mounted with antifade mounting medium with DAPI (Solarbio Science & Technology Co., Ltd, Beijing, China). Images were captured by the laser scanning confocal microscope (ZEISS LSM710, Oberkochen, Germany).

To examine internalized antibodies and the trafficking to lysosomes, the cells were incubated with 5 μg·mL^−1^ anti‐CD30‐LDP at 37 °C for 24 h in the presence of 50 μg·mL^−1^ leupeptin (Amresco, Solon, OH, USA) and 25 μg·mL^−1^ pepstatin (Amresco) to inhibit lysosomal degradation. Cells were then washed and centrifuged to the glass slides using the cytospin and fixed with 4% paraformaldehyde for 10 min, permeabilized with 0.2% Triton X‐100 in PBS for 5 min at room temperature. After blocking the nonspecific sites with 5% normal goat serum, cells were incubated with rabbit anti‐LAMP‐1 antibody (Cell Signaling Technology, Danvers, MA, USA) overnight at 4 °C followed by anti‐rabbit Alexa Fluor 555 to label the LAMP‐1 on lysosomes and anti‐human Alexa Fluor 488 antibodies to label anti‐CD30‐LDP, respectively. The slides were blocked with antifade mounting medium with DAPI. Images were captured by the laser scanning confocal microscope (ZEISS LSM710).

### 2.7 *In vivo* imaging of fluorescein‐labeled anti‐CD30‐LDP

*In vivo* tumor‐targeting ability of anti‐CD30‐LDP was investigated using Karpas299 and L540 xenograft tumor models in NOD/SCID mice. Anti‐CD30‐LDP and the free LDP (provided by our laboratory) were labeled with the DyLight 680 Dyes (Thermo Fisher Scientific) according to the manufacturer's instruction and then were injected into the tail vein at a dose of 20 mg·kg^−1^ when the solid tumors reached 200–300 mm^3^, respectively. The mice were placed in the imaging chamber of the Xenogen IVIS‐200 system (Xenogen Inc., Alameda, CA, USA) for *in vivo* distribution observation at a series of time points after anesthetized by isoflurane. The images were also analyzed by the living image software (Caliper Life Science, Hopkinton, MA, USA).

### 2.8 Preparation of the anti‐CD30‐LDM

The chromophore AE of LDM was separated through a C4 column (150 × 10 mm; Phenomenex, Torrance, CA, USA) by HPLC. Then, the AE‐containing solution was mixed with the anti‐CD30‐LDP solution at a 1 : 3 molecular ratio and incubated at 4 °C for 12 h by gently shaking to form the enediyne‐integrated ADC anti‐CD30‐LDM. Next, free AE was removed by ultrafiltration centrifugation. The composition of the ADC was finally confirmed by reverse‐phase HPLC using a C4 column (250 × 4.6 mm; Phenomenex).

### 2.9 *In vitro* cytotoxicity assay

The cytotoxicity of the enediyne‐integrated anti‐CD30‐LDM was analyzed by the Cell Counting Kit‐8 (CCK‐8; Dojindo). Briefly, the different lymphoma cell lines were seeded at 2.5 × 10^4^ cells in 100 μL complete medium into 96‐well plates and incubated at 37 °C with 5% CO_2_ for 2 h. Then, anti‐CD30‐LDM and LDM were added in triplicate at different concentrations with 100 μL medium, respectively. After 48‐h incubation, 20 μL CCK‐8 reagent was added and incubated for 1 h. The absorbance was measured at 450 nm using a microplate reader. Untreated cells served as control. The relative cell survival (%) was calculated using the following formula: [(*A*_sample_ − *A*_blank_)/(*A*_control_ − *A*_blank_)] × 100%. The 50% inhibitory concentration (IC_50_) of the samples was calculated by spss software (IBM SPSS, Chicago, IL, USA).

### 2.10 Cell cycle arrest and apoptosis analysis

Flow cytometry was used to analyze the effects of anti‐CD30‐LDM on Karpas299 and L540 cell lines. For the cell apoptosis analysis, 2 × 10^5^ cells per well were plated in 6‐well culture plates and incubated at 37 °C for 2 h. Then, anti‐CD30‐LDM with different concentration was added into the wells for 24 h, and untreated cells served as control. Apoptosis was detected following the manufacturer's instruction of Annexin V‐FITC apoptosis kit (Dojindo) and finally analyzed with FACS Calibur. For the cell cycle arrest analysis, the cells were treated as described above and following the instructions of Cell Cycle and Apoptosis Analysis Kit (Beyotime Biotechnology, Shanghai, China). Then, the samples were detected with FACS Calibur.

### 2.11 Western blot analysis

Cells were treated with the indicated drugs for different time periods, then washed twice with ice‐cold PBS, and lysed in RIPA tissue/cell lysis buffer (Solarbio Science & Technology Co., Ltd). The concentrations of protein samples were quantified using a BCA protein assay kit (Thermo Fisher Scientific). The samples were electrophoretically separated on SDS/PAGE and transferred to PVDF membranes. After being blocked, the membranes were then probed with specific antibodies. Protein bands were detected with an enhanced chemiluminescence kit (Merck Millipore, Darmstadt, Germany). The following antibodies were used anti‐p53 (Cell Signaling Technology, #9282), anti‐p21^WAP1/CIP1^ (Cell Signaling Technology, #2947), antiphospho‐p53 (Ser15) (Cell Signaling Technology, #9284), anticleaved caspase 3 (Cell Signaling Technology, #9664), anti‐PARP (Cell Signaling Technology, #9532), anti‐β‐tubulin (Cell Signaling Technology, #2128), anti‐CD30 (Abcam, Cambridge, MA, USA, ab134080), and anti‐β‐actin (ZSGQ‐BIO, Beijing, China).

### 2.12 Caspase‐3/7 activity

Suspended cells were plated at 2 × 10^4^ per well in a 96‐well white‐walled plate and treated with the indicated drugs for 12 h before being assayed with the caspase‐glo 3/7 Assay Kit (Promega, Madison, WI, USA) according to the manufacturer's recommendation. Briefly, the caspase‐glo 3/7 substrate was dissolved with the relevant buffer to form the caspase‐glo 3/7 reagent, and then, equal volume of reagent was added to samples and incubated for 30 min at room temperature. The luminescent signal was read with a microplate reader (SpectraMax i3x, Molecular Devices, Santa Clara, CA, USA).

### 2.13 *In vivo* therapeutic efficacy

Female NOD/SCID mice were purchased from Beijing Vital River Laboratory Animal Technology Co., Ltd. (Beijing, China), and housed under specific pathogen‐free condition. All animal experiments were approved by the Institutional Animal Care and Use Committee of the Institute of Medicinal Biotechnology, Chinese Academy of Medical Sciences. 6 × 10^6^ Karpas299 cells suspended in 200 μL PBS were injected subcutaneously into the right flank of 6‐ to 8‐week‐old mice. When the tumor volume reached 100 mm^3^, mice were randomized into six groups (*n* = 6 per group) and treated intravenously with various doses of anti‐CD30‐LDM, or 0.6 mg·kg^−1^ anti‐CD30 antibody, or 0.045 mg·kg^−1^ LDM, respectively, every 7 days for a total of two injections. The control group was given PBS only. Tumors were measured twice a week with a caliper, and tumor volumes were determined using the formula: (length × width^2^)/2. The inhibition rate of tumor growth was calculated as [1 − (tumor volume_treated final_ − tumor volume_treated initial_)/(tumor volume_control final_ − tumor volume_control initial_)] × 100%. At the end of the experiment, mice were euthanized. Subcutaneous tumors and various organs were harvested and fixed in 10% formalin for hematoxylin and eosin (H&E) staining and immunohistochemical analysis.

### 2.14 Statistical analysis

Results of all experiment were presented as mean values ± standard deviations (SD) by the graphpad prism 5 software (GraphPad Software, Inc., San Diego, CA, USA). IC_50_ values were determined by nonlinear regression analysis of concentration–response curves using spss 16.0. The statistical significance between two groups was determined using two‐way ANOVA followed by Student's *t*‐test. For all tests, *P*‐values < 0.05 were considered statistically significant.

## 3 Results

### 3.1 Construction, expression, purification, and characterization of fusion proteins

The goal of this study was to generate a novel enediyne‐integrated ADC, named anti‐CD30‐LDM, which composed of a chimeric antibody directed against CD30 and the extremely potent cytotoxic agent LDM (Fig. S1A). First, we constructed an expression vector of the fusion protein. The plasmids encoding for anti‐CD30‐LDP and anti‐CD30 antibody were obtained by genetic engineering (Fig. S1B). After transfection, selection, and purification, the proteins were analyzed by SDS/PAGE gels. Under reducing conditions, the heavy chains of anti‐CD30 antibody and anti‐CD30‐LDP migrated as the same MW of 50 kDa, while the light chains showed a MW of approximately 25 and 36 kDa, respectively, indicating the latter contained a LDP head. The nonreducing SDS/PAGE indicated that the heavy and light chains were properly assembled to generate recombinant proteins with the MW approximately 150 kDa (anti‐CD30 antibody) and 172 kDa (anti‐CD30‐LDP), respectively (Fig. S2A). Determined by HPLC, the purity of anti‐CD30‐LDP and anti‐CD30 antibody was more than 98% after affinity chromatography (Fig. S2B,C).

### 3.2 Anti‐CD30‐LDP had high‐affinity binding to recombinant human CD30 antigen

**Figure 1:**
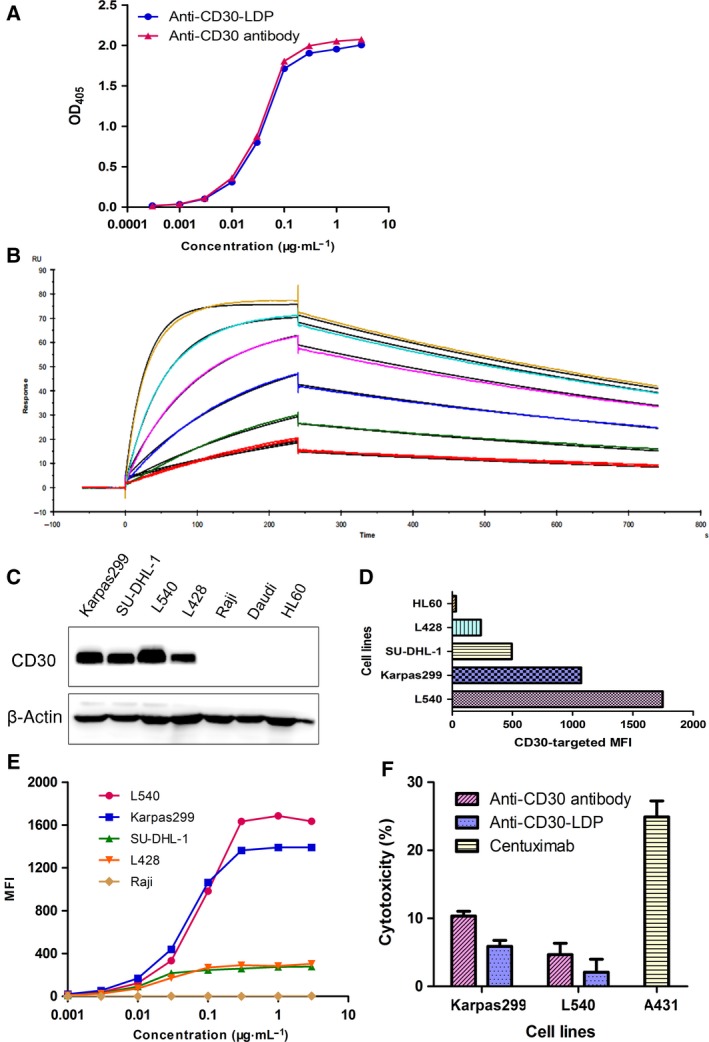
Anti‐CD30‐LDP retains binding and functional properties of parent anti‐CD30 antibody *in vitro*. (A) Binding activity of anti‐CD30‐LDP and anti‐CD30 antibody to recombinant human CD30 by ELISA. (B) Surface plasmon resonance (SPR) sensorgrams of anti‐CD30‐LDP binding with CD30 antigen. Various concentrations of antigen (human recombinant CD30, 2.03‐65 nm) were applied on CM5 chip with immobilized anti‐CD30‐LDP fusion protein. Each sensorgram represents different concentrations of CD30. The CD30 at 2.03 nm was injected twice to verify reproducibility. (C) Western blot analysis of CD30 expression levels on different cancer cells. (D) Binding activity to different cell lines. The cells were incubated with anti‐CD30‐LDP of 10 μg·mL^−1^ and FITC‐conjugated anti‐human Fc second antibody, and then, cell‐bound fluorescence was determined by FACS analysis. The horizontal axis represents the values of MFI. (E) Binding curves of increasing concentrations of anti‐CD30‐LDP (0.001‐3 μg·mL^−1^) to different cancer cells by FACS analysis. The vertical axis represents the MFI values. (F) *In vitro* ADCC analysis of anti‐CD30‐LDP and anti‐CD30 antibody. The ADCC assay was performed using PBMCs as effector cells and Karpas299 or L540 cells as target cells at a ratio of 40 : 1. The concentration of anti‐CD30‐LDP was 10 μg·mL^−1^. Meanwhile, the cetuximab and the target cell line A431 were used as a positive control.

The antigen‐binding properties of anti‐CD30‐LDP are crucial for its *in vitro* and *in vivo* activity. The binding of both the fusion protein and their parent antibody to recombinant human CD30 was assayed by ELISA. Anti‐CD30‐LDP showed a saturable dose–response curve similar with the parent anti‐CD30 antibody (Fig. 1A), which indicated that the fusion of LDP to the antibody had minimal impact on the antigen‐binding activity.

To precisely determine the affinity of anti‐CD30‐LDP and its parental anti‐CD30 antibody to antigen, we employed SPR experiment. The binding events of anti‐CD30‐LDP to CD30 were monitored, and the sensorgrams are concentration‐dependent, as expected (Fig. 1B). The antigen–antibody interaction demonstrated that anti‐CD30‐LDP and anti‐CD30 antibody had potent and comparable binding affinity to recombinant CD30 with the apparent equilibrium dissociation constant (*K*_D_) showing 2.08 and 2.38 nm, respectively (Table 1). The results provided further evidence that the LDP moiety had no adverse effects on antibody affinity for anti‐CD30‐LDP.

**Table 1:** Biosensor kinetics and affinity to CD30 by SPR

Antibodies	*k*_a_ (M^−1^·s^−1^)	*k*_d_ (s^−1^)	*K*_D_ (m)
Anti‐CD30‐LDP	5.32 × 10^5^	1.10 × 10^−3^	2.08 × 10^−9^
Anti‐CD30 antibody	4.61 × 10^5^	1.10 × 10^−3^	2.38 × 10^−9^

*k*_a,_ kinetic association rate constant; *k*_d,_ kinetic dissociation rate constant; *K*_D,_ equilibrium dissociation constant.

### 3.3 Anti‐CD30‐LDP can efficiently bind to CD30‐positive cell lines

**Figure 2:**
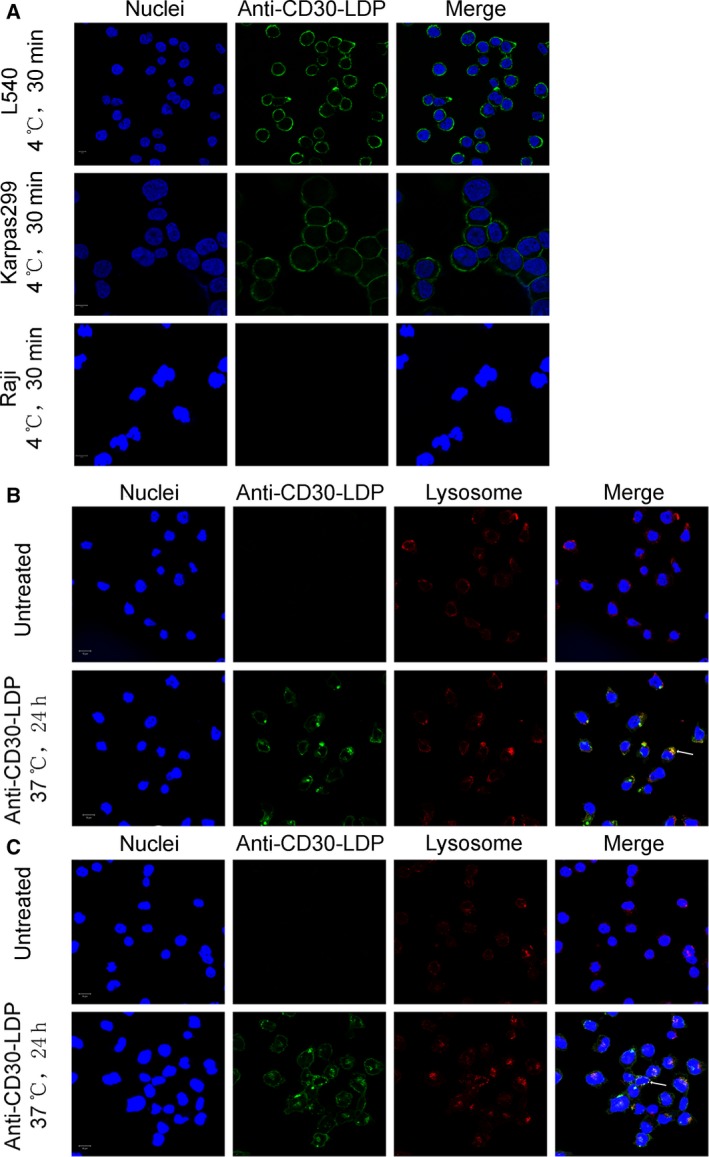
Receptor‐mediated internalization of anti‐CD30‐LDP by CD30^+^ lymphoma cells. Cells were treated at 4 °C with anti‐CD30‐LDP labeled with Alexa Fluor 488‐conjugated anti‐human IgG. Anti‐CD30‐LDP bound to CD30‐positive cell line L540 and Karpas299, while no signal was detected on CD30‐negative cell line Raji (A). Anti‐CD30‐LDP was internalized and trafficked to the lysosomes in L540 (B) and Karpas299 (C) cells at 37 °C for 24 h. Cell surface or intracellular anti‐CD30‐LDP was visualized by immunofluorescence confocal microscopy. Anti‐CD30‐LDP is shown in green, the lysosomal marker Lamp‐1 is shown in red, and the Hoechst‐stained nuclei are in blue. Colocalization of signals for anti‐CD30‐LDP with Lamp‐1 is shown in yellow. The data shown were representative of three independent experiments. Scale bar = 10 μm.

To mediate cytotoxicity, anti‐CD30‐LDP fusion protein must bind to the native antigen on cancer cells. First, we detected the levels of CD30 expression on different cancer cells by western blot. As shown in Fig. 1C, both the HD cell lines (L540 and L428) and ALCL cell lines (Karpas299 and SU‐DHL‐1) expressed high levels of CD30, but there was higher expression on L540 and Karpas299 cells. In contrast, no CD30 expression was found on Raji, Daudi, and HL60 cells. In addition, the FACS assays showed that the binding signals of anti‐CD30‐LDP under saturation state were positively correlated with the expression levels of CD30 on different cancer cells, which supported the results of Fig. 2C and indicated that the fusion protein can bind to the native CD30 antigen (Fig. 1D). The differences in binding affinity among various cell lines were further investigated by FACS. Measurement of the binding of anti‐CD30‐LDP after incubation at different concentrations with identical numbers of cells revealed a saturable dose–response curve (Fig. 1E). All four CD30^+^ cell lines showed saturable binding kinetics, and there was higher mean fluorescence intensity (MFI) for which with high levels of CD30 expression at the same concentration. Moreover, the observation that the half‐maximal binding occurred at the similar concentration (approximate 0.05 μg·mL^−1^) suggested that the affinity of anti‐CD30‐LDP toward native CD30 is similar for all tested cell lines. There was no binding between anti‐CD30‐LDP and CD30^−^ Raji cells. The results demonstrated that anti‐CD30‐LDP not only recognized immobilized antigen mentioned above, but also had high‐affinity and specific binding with native CD30 on the surface of lymphoma cells.

### 3.4 The ADCC effect of anti‐CD30‐LDP against HL or ALCL cells

Antibody‐dependent cellular cytotoxicity has been described as a major efficacy of action for immunotherapy with therapeutic antibodies relying on their constant region (Fc domain) ability (Kashyap *et al*., 2017). To characterize the Fc‐mediated ADCC activity of anti‐CD30‐LDP or anti‐CD30 antibody, L540 or Karpas299 cells were incubated with the antibody in the presence of PBMCs. However, unexpectedly, both anti‐CD30 antibody and anti‐CD30‐LDP had moderate ADCC activity compared with cetuximab. Meanwhile, ADCC activity of anti‐CD30‐LDP was minimal compared to anti‐CD30 antibody (Fig. 1F). Taken together, these results indicated that our fusion protein may not rely on ADCC as their main mechanism of cytotoxicity.

### 3.5 CD30‐mediated endocytosis and intracellular trafficking

To kill cancer cells, the anti‐CD30‐LDM needs to be internalized and released into the cytoplasm, so we detected the internalization and trafficking of anti‐CD30‐LDP via fluorescently labeled antibody in Karpas299 and L540 cell lines with laser scanning confocal microscope. Lysosomal compartments were visualized by staining with LAMP‐1 antibody. Initially, the proteins were incubated with the cells at 4 °C, a temperature that allowed binding but not internalization (Maruani *et al*., 2015). Anti‐CD30‐LDP staining was uniformly localized on the plasma membrane, which is particularly visible around the cell shape on Karpas299 and L540 cells, while there was no fluorescence localized on the plasma membrane for CD30^−^ Raji cells (Fig. 2A). On the other hand, after incubation at 37 °C, it occurred capping and punctate staining for anti‐CD30‐LDP in the cells and reduced staining on the cell surface (Fig. 2B,C). Meanwhile, the intracellular protein signals colocalized with the signals of LAMP‐1, indicating that anti‐CD30‐LDP was internalized and transported to the lysosomes.

### 3.6 *In vivo* imaging of fluorescein‐labeled anti‐CD30‐LDP

**Figure 3:**
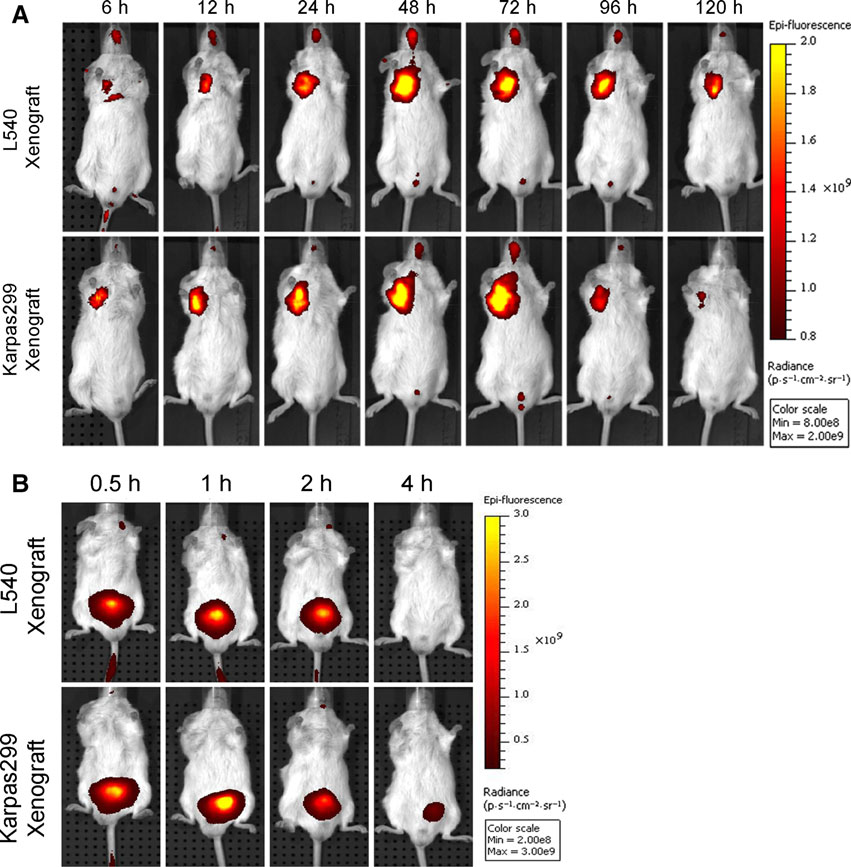
Targeting of fluorescently labeled proteins to established L540 and Karpas299 xenografts in NOD/SCID mice. Anti‐CD30‐LDP (A) or free LDP (B) was labeled with DyLight 680 and injected into tumor‐bearing NOD/SCID mice through tail veins at 20 mg·kg^−1^, respectively. *In vivo* fluorescence images were taken at appointed time. Color scale represents photons·s^−1^·cm^−2^·steradian^−1^.

*In vivo* distribution and tumor‐targeting capability of anti‐CD30‐LDP were observed in NOD/SCID mice bearing the CD30‐positive Karpas299 or L540 xenografts via an optical molecular imaging system. The results demonstrated that there was a strong and selective accumulation in CD30‐positive tumors in the case of the anti‐CD30‐LDP fusion protein (Fig. 3A). After *i.v*. administration of DyLight 680 labeled anti‐CD30‐LDP, the fluorescence signal was clearly visualized in the tumor sites within 6 h. Then, an increasing tumor uptake was detected at 12 and 24 h and maximum at 48, whereas the signal intensity decreased by 96 h and remained detectable for more than 120 h. In addition, for the free LDP protein, it showed a rapid renal clearance in half an hour postinjection and no detectable localization in any of the two tumor types (Fig. 3B). These results indicated that anti‐CD30‐LDP could target to the tumors effectively *in vivo* and carry LDM to tumor sites.

### 3.7 The assembly of enediyne‐integrated anti‐CD30‐LDM

**Figure 4:**
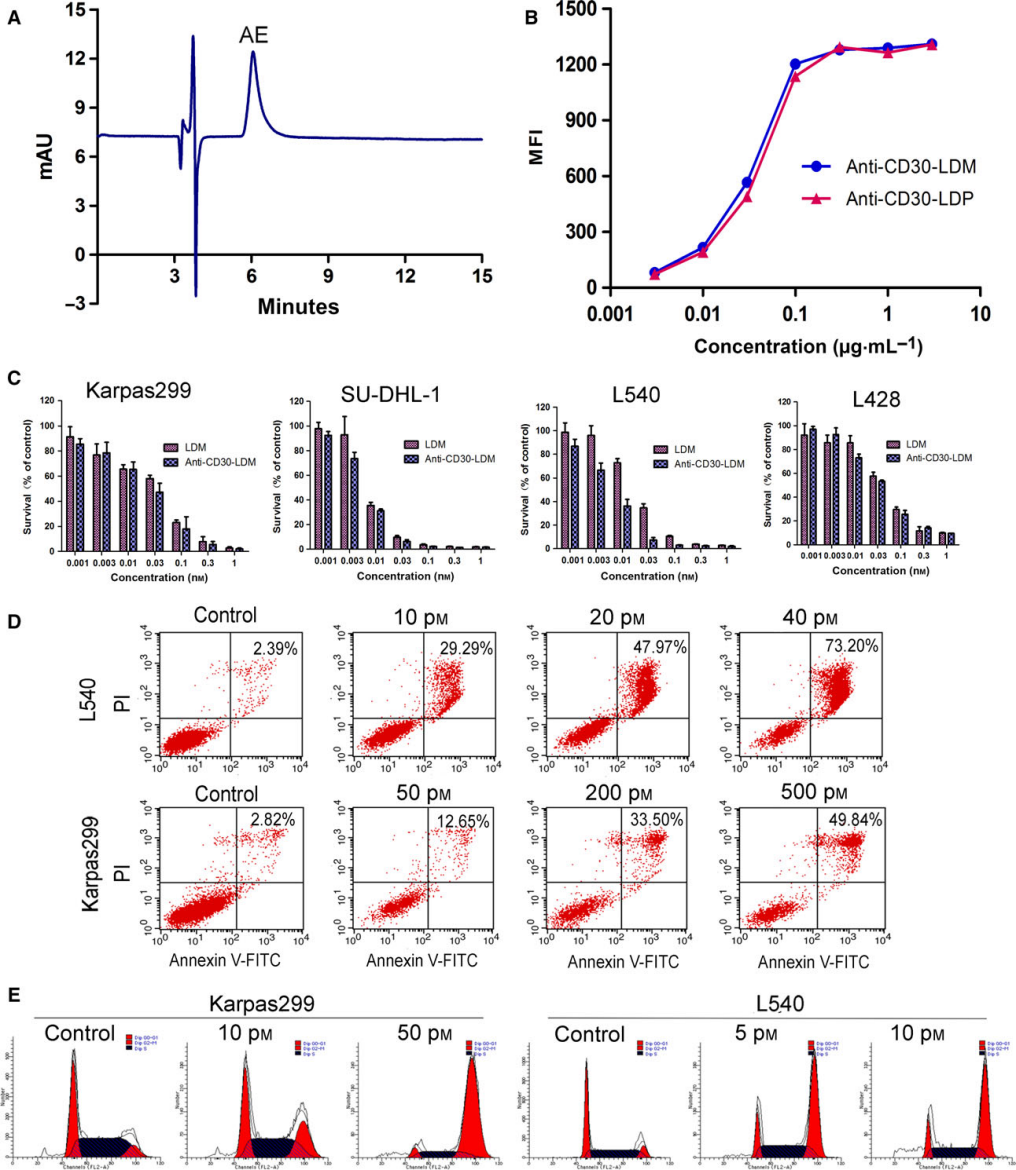
The characterization and *in vitro* activity of anti‐CD30‐LDM. (A) Reverse‐phase HPLC analysis of enediyne‐integrated anti‐CD30‐LDM using a Vydac C4 300A column at 340 nm. (B) Binding affinity of anti‐CD30‐LDM and anti‐CD30‐LDP to Karpas299 cells by FACS. (C) Cell viability assay. Karpas299, SU‐DHL‐1, L540, and L428 cell lines were treated with anti‐CD30‐LDM and LDM in a series of concentrations (0.001–1 nm) for 48 h. Cell viability was tested by Cell Counting Kit‐8 (CCK‐8). Results are the mean values ± SD of three replicates. (D) Flow cytometry analysis of apoptosis of L540 or Karpas299 cells treated with increasing concentrations of anti‐CD30‐LDM for 24 h, respectively. (E) Cell cycle arrest assay of Karpas299 or L540 cells by flow cytometry. Cells were treated with indicated concentrations of anti‐CD30‐LDM for 24 h.

We had demonstrated that anti‐CD30‐LDP had exciting affinity and specificity to CD30, which was crucial for performing targeted cytotoxicity. Then, the enediyne‐integrated fusion protein of anti‐CD30‐LDM was prepared by assembling the active enediyne chromophore AE of LDM into anti‐CD30‐LDP. The remaining free AE was removed by ultrafiltration, and the permeate was detected by reverse‐phase HPLC until there was no peak for AE shown (Fig. S3). The anti‐CD30‐LDM solution was examined using a Vydac C4 300A column, and the results showed that there was a characteristic peak of AE at 340 nm which indicated that the chromophore AE was assembled into anti‐CD30‐LDP fusion protein successfully (Fig. 4A). Additionally, the procedure of assembly had almost no influence on the affinity of antibodies because the binding activity of anti‐CD30‐LDM was close to anti‐CD30‐LDP on Karpas299 cells by FACS (Fig. 4B).

### 3.8 *In vitro* cytotoxicity of anti‐CD30‐LDM

The cytotoxic efficacy of anti‐CD30‐LDM to the HL cell lines L540 and L428 as well as the ALCL cell lines Karpas299 and SU‐DHL‐1 was tested *in vitro,* while the LDM was used as positive control. Both anti‐CD30‐LDM and free LDM effectively killed the tested cell lines (Fig. 4C), with IC_50_ values (the 50% inhibiting concentration) ranging from 0.54 × 10^−11^ to 3.74 × 10^−11^ m (Table 2). We also investigated the cytotoxicity of the unintegrated anti‐CD30‐LDP, and the results showed no or minimal effect on cell viability under the same concentrations of anti‐CD30‐LDM (Fig. S4A,B). Thus, we can conclude that the cytotoxicity of anti‐CD30‐LDM was mainly due to the action of AE molecule. The apoptosis assay showed that the ratio of late‐stage apoptosis of Karpas299 and L540 cells was increased in a concentration‐dependent manner when treated with anti‐CD30‐LDM (Fig. 4D). Meanwhile, anti‐CD30‐LDM could lead to G2/M cell cycle arrest and cell apoptosis (Fig. 4E).

**Table 2:** IC_50_ values of anti‐CD30‐LDM and LDM to various cancer cell lines

Cell lines	IC_50_ (×10^−11^ m)	
	Anti‐CD30‐LDM	LDM
L540	0.54 ± 0.13	1.97 ± 0.19
Karpas299	1.73 ± 0.51	2.28 ± 0.30
SU‐DHL‐1	0.59 ± 0.07	0.63 ± 0.14
L428	3.74 ± 0.16	4.17 ± 1.48

### 3.9 Anti‐CD30‐LDM induced p53‐ and caspase‐3/7‐independent apoptosis in HL and ALCL

**Figure 5:**
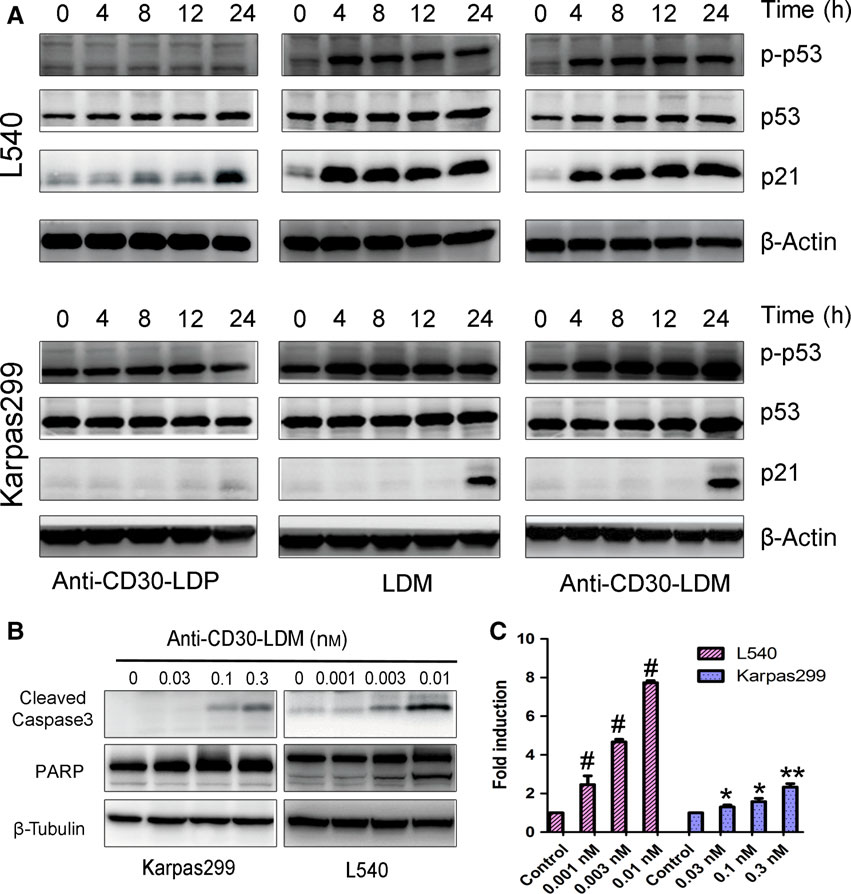
Molecular mechanism of anti‐CD30‐LDM‐mediated cell apoptosis. Western blot presented the levels of apoptosis‐associated proteins (A, B). (A) Cells were exposed to 0.03 nm anti‐CD30‐LDP/LDM/anti‐CD30‐LDM, respectively, and samples were collected at the appointed time to analyze the levels of p21^WAP1/CIP1^, p53, and p‐p53. β‐Actin was used as a loading control. (B) Cells were exposed to the appointed concentration of anti‐CD30‐LDM for 12 h, and samples were collected to analyzes the levels of cleaved‐caspase 3 and PARP. β‐Tubulin was used as a loading control. (C) Induction of caspase‐3/7 activity in Karpas299 or L540 cells treated with anti‐CD30‐LDM for 12 h. Fold induction in caspase‐3/7 activity was determined as described in Materials and methods. Means and SDs of triplicate experiments are shown (*P < 0.01, **P < 0.001, ^#^ *P* < 0.0001).

We investigated the molecular mechanism of cell death induced by anti‐CD30‐LDM. It is well known that the tumor suppressor p53 plays a key role in both of cell cycle and cell proliferation (Chen *et al*., 2007). We first tested whether anti‐CD30‐LDM can induce p53‐mediated apoptosis in L540 and Karpas299 cells. Immunoblotting analysis was executed to evaluate the effect of anti‐CD30‐LDM on the expression of apoptosis‐associated proteins involving p53, p‐p53 (Ser15), and p21^WAP1/CIP1^ (Fig. 5A). As shown, the expression of p53 was improved slightly in both cell lines. Compared with anti‐CD30‐LDP, higher levels of p53 phosphorylation at serine 15 were observed in cells treated with anti‐CD30‐LDM and LDM. As reported, upon DNA damage or during cell cycle arrest, p53 is phosphorylated and p21 transcription upregulated (Mytych *et al*., 2017; Younger and Rinn, 2017); and p21 has been also proposed to contribute to G2 arrest in response to DNA damage (Cazzalini *et al*., 2010). The expression levels of p21^WAP1/CIP1^ significantly increased in cells treated with LDM and anti‐CD30‐LDM in a time‐dependent manner. In the apoptotic cells, activation of caspase 3 and inactivation of poly (ADP‐ribose) polymerase (PARP) occur where DNA damage is extensive. So, we detected whether the expression levels of cleaved‐caspase 3 and PARP change in Karpas299 and L540 cells treated with anti‐CD30‐LDM. The results showed that caspase 3 was activated (cleaved) and PARP was inactivated by caspase 3 cleavage in a concentration‐dependent manner (Fig. 5B). We next detected the activation levels of caspase‐3/7 in L540 and Karpas299 cells. As shown, anti‐CD30‐LDM led to marked activation of caspase‐3/7 in both cell lines (Fig. 5C). Collectively, these results explained that antiproliferative effects observed with anti‐CD30‐LDM could be accounted for, at least in part, by the p53‐ and caspase‐dependent signaling pathway.

### 3.10 *In vivo* therapeutic efficacy

**Figure 6:**
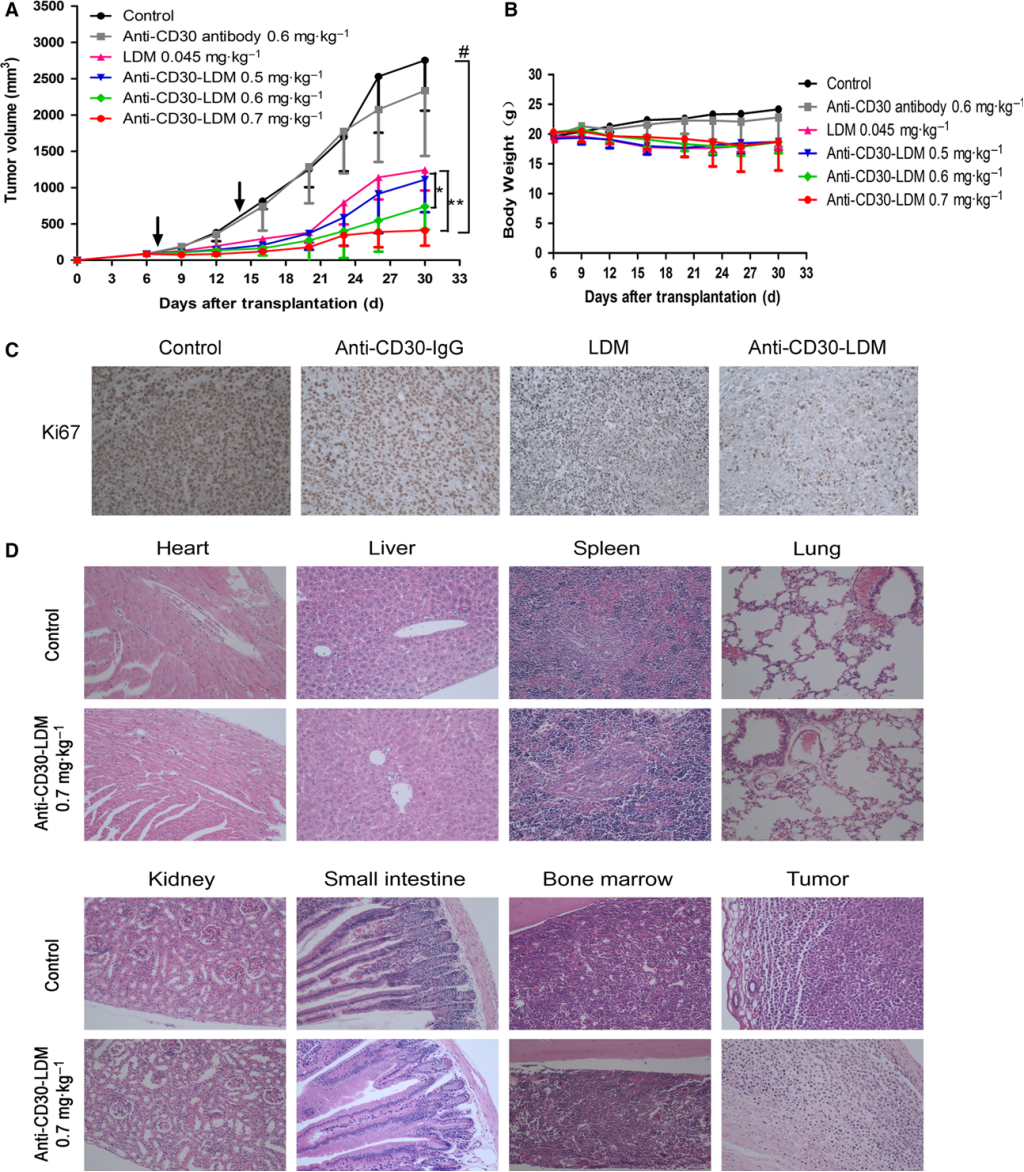
*In vivo* efficacy of anti‐CD30‐LDM against Karpas299 xenograft model. (A) The antitumor effects of anti‐CD30‐LDM in NOD/SCID mice bearing Karpas299 xenografts (*n* = 6). The agents were administered at doses indicated in the figure on a Q7D × 2 schedule, and arrows indicate days of administration. *P < 0.05 and **P < 0.001 compared with the LDM group; ^#^ *P* < 0.0001, compared with the control. (B) Body weight change of Karpas299 xenograft‐bearing mice. (C) Tumor sections were stained for Ki‐67 as the representative microscopic images (×200) shown. (D) H&E staining of various organs and tumors of Karpas299 xenograft‐bearing mice treated with anti‐CD30‐LDM at a dose of 0.7 mg·kg^−1^. No toxico‐pathological changes were found in the organs indicated in the figure, compared with the control (×200).

To evaluate whether anti‐CD30‐LDM had therapeutic potential *in viv*o, we established the Karpas299 xenograft model in NOD/SCID mice. Remarkably, anti‐CD30‐LDM inhibited tumor growth in a dose‐dependent manner, while the unconjugated antibody had minimal effect (Fig. 6A). Free LDM at the maximum tolerated dosages of 0.045 mg·kg^−1^ inhibited tumor growth by 56.73%, while anti‐CD30‐LDM at dosages of 0.5, 0.6, 0.7 mg·kg^−1^ resulted in 61.58%, 75.47%, 87.76% inhibition, respectively. During the experiment, there were no death and no significant adverse reactions, such as lethargy and anorexia, and the changes of body weight were in acceptable range (Fig. 6B).

Next, paraffin sections of tumors were observed for the effect of anti‐CD30‐LDM on mitotic index (Ki‐67) using immunohistochemical methods. Ki‐67‐positive cells were apparently reduced in anti‐CD30‐LDM‐treated groups, indicating less proliferation compared with the groups of control, anti‐CD30 antibody, and LDM (Fig. 6C). Moreover, as shown in Fig. 6D, *in vivo* toxicity and efficacy of anti‐CD30‐LDM were also evaluated by tissue morphological analysis (H&E staining). On the one hand, no significant toxico‐pathological changes were found in the heart, liver, spleen, lung, kidney, small intestine, and bone marrow of mice treated with anti‐CD30‐LDM at the large dosage of 0.7 mg·kg^−1^, which suggested that those given dosages were well tolerated. On the other hand, the tumor of control group displayed more round or polygonal neoplastic cells with blue nuclei and clear cytoplasm, while the treated group showed more dead cells with pyknotic nuclei and not clear cytoplasm. These results supported the favorable antitumor activity of anti‐CD30‐LDM.

## 4 Discussion

CD30 has been implicated as an excellent potential target for the treatment of HL and ALCL with the data presented previously (Fanale *et al*., 2012; Francisco *et al*., 2003; Gopal *et al*., 2015; Pro *et al*., 2012). The MMAE‐conjugated anti‐CD30 ADC BV has shown exciting response rates in both relapsed/refractory HL and ALCL (Chen *et al*., 2016; Pro *et al*., 2012). Despite high response rates, the majority of the observed responses are partial and short‐lived (Younes *et al*., 2012). Patients with relapsed or refractory HL treated with BV have a median progression‐free survival (PFS) of less than 10 months, and the 5‐year PFS rate was 22% (Chen *et al*., 2016; Gopal *et al*., 2015; Younes *et al*., 2012). In a randomized, double‐blind, placebo‐controlled, phase 3 trial, the most frequent adverse event in BV treatment was peripheral sensory neuropathy in more than 50% patients (Moskowitz *et al*., 2015). Besides, MMAE resistance has been reported as one of the BV resistance mechanisms explored from patients who progressed on BV therapy (Chen *et al*., 2015). This highlights the need to study new ADCs and payloads which have different mechanisms of action from MMAE for the therapy of CD30^+^ lymphoma. In this study, we generated a novel type of ADC anti‐CD30‐LDM by integrating the LDM‐derived enediyne molecule AE into the recombinant fusion protein anti‐CD30‐LDP. Being a member of enediyne‐containing antitumor antibiotic family, LDM has been developed by our institute and is currently in phase II clinical trials. The potential clinical value of enediyne antibiotics is also highlighted by the recent approval of Besponsa and Mylotarg, immunoconjugates which used calicheamicin, another member of enediyne‐containing family, as a cytotoxic payload (Godwin *et al*., 2017; Lamb, 2017). Studies have reported that LDM inhibits DNA synthesis, causes cellular DNA breakage, and subsequently blocks the progression of cells at G2/M phase with IC_50_ values in the subnanomolar range (Shao and Zhen, 2008). Our data presented that anti‐CD30‐LDM showed similar mechanism of action as LDM. It induced cell cycle arrests and apoptosis through altering the expression and modification status of p53 as well as the expression of p21^WAF1/CIP1^. There is good evidence that p53 and p21 are involved in blocking cells at G2/M and DNA damage (Cazzalini *et al*., 2010). This study demonstrated that anti‐CD30‐LDM and LDM increased the expression levels of p‐p53, p53, and p21, while anti‐CD30‐LDP had minimal effect. The caspase‐associated pathway was also activated in the apoptotic process by the activation of caspase 3 and inactivation of PARP when treated with anti‐CD30‐LDM. In addition, in contrast to the reported ‘warhead’ agent MMAE, lidamycin does not block the polymerization of tubulin but exerts its cytotoxic effects through inducing DNA damage. Therefore, we speculated that anti‐CD30‐LDM might have the potential to overcome resistance induced by BV.

Current methods for linking payloads to the antibodies in ADCs predominantly utilize conventional bioconjugation strategies through the sites of the amino groups of lysine residues or thiol groups generated by the reduction of hinge region disulfides (Zhou *et al*., 2014). Unfortunately, the random conjugation processes produce a diverse population of ADCs with a wide distribution of drug molecules per antibody (Wang *et al*., 2005). The structural heterogeneity in overall charge can impact solubility, stability, efficacy, and potentially increase off‐target toxicity (Junutula *et al*., 2008). Moreover, there were studies concluded that ADCs loaded with 2–4 drug molecules per mAb achieved the best balance between slow clearance and maximal potency (Peters and Brown, 2015). Furthermore, research had demonstrated that ADCs with a payload conjugated to the light chain of the mAb were shown to perform significantly higher *in vivo* efficacy compared with those conjugated to the heavy chain (Shen *et al*., 2012). On the other hand, noncleavable linkers are intended to be more stable in the bloodstream and extracellular space, because ADCs release their toxins relying on the internalization and lysosomal degradation (DeVay *et al*., 2017; Peters and Brown, 2015; Ritchie *et al*., 2013). Therefore, ADC efficacy depends in part on antibody–antigen interaction at the cell surface triggering the internalization and lysosomal trafficking. In the present work, the LDM‐derived apoprotein LDP was fused with the N‐terminal of light chain of the anti‐CD30 antibody through a noncleavable linker, so the resulting anti‐CD30‐LDM contained exactly two LDM molecules per antibody. Furthermore, the results showed that fusion protein anti‐CD30‐LDP has high‐binding activity to CD30 antigen and can be internalized into CD30^+^ tumor cells. The fusion of LDP to anti‐CD30 antibody had no effect on the activity of parent anti‐CD30 antibody. Anti‐CD30‐LDM displayed potent cytotoxicity to cancer cells *in vitro*. These results suggested that our design for generating novel types of ADC is both rational and feasible.

In this study, we generated the new ADC by a novel two‐step method according to the attractive property of the lidamycin which can be dissociated and reconstituted *in vitro* (Tanaka *et al*., 2001). First, the fusion protein anti‐CD30‐LDP, an anti‐CD30 antibody fused with the LDP of lidamycin, was generated through genetic engineering and expressed in CHO cell culture. Second, anti‐CD30‐LDM was prepared by integrating the enediyne chromophore AE into the fusion protein anti‐CD30‐LDP through molecular reconstitution. Compared with complex chemical processes such as the synthetic of linkers and the modification of antibodies and cytotoxic drugs, which is essential for the generation of ADCs, the present process is an alternative way to generate a new type of ADC. Of importance, the study has shown that the integration of enediyne chromophore AE into anti‐CD30‐LDP exerted no effect on its antibody properties; in addition, AE retained its extremely potent cytotoxicity against the target cells. Therefore, the present two‐step protocol can serve as a feasible technical platform to construct a new type of ADC.

In our previous work, we produced the fusion protein dFv‐LDP‐AE‐containing antigelatinases tandem scFv and lidamycin (Zhong *et al*., 2010). However, it was expressed in *Escherichia coli* and required tedious refolding from *E. coli* inclusion bodies and showed relatively low affinity. The present study is the first one which uses an intact antibody to fuse with lidamycin and then expressed it in CHO cells successfully. Our data demonstrated that the binding affinity had been significantly improved and the tumor retention time of more than 5 days *in vivo*, which was much longer than that of 6 h for dFv‐LDP‐AE in previous studies. The *in vivo* antitumor study further confirmed that anti‐CD30‐LDM significantly improved the antitumor efficiency compared with naked anti‐CD30 antibody or free lidamycin alone. Moreover, anti‐CD30‐LDM exhibited favorable safety profile because no obvious lesions were observed in various organs of treated mice at therapeutic doses by histopathological examination. In brief, the fusion of LDM to an anti‐CD30 mAb improved the tolerability and tumor‐therapeutic efficacy of LDM in NOD/SCID mice. Building on the long‐standing concept that immunity plays an important role in cancer pathogenesis, we had detected the influence of anti‐CD30‐LDM and anti‐CD30 antibody on the expression of programmed cell death‐1 ligand 1 (PD‐L1) in Karpas299 and L540 cells. Interestingly, both the proteins augment PD‐L1 presentation (Fig. S5A,B), which indicated that combining targeted therapy with immunotherapy may achieve greater antitumor effects than that achieved with either monotherapy. So in future studies, we will investigate whether there is any synergistic antitumor activity of anti‐CD30‐LDM combined with immunotherapy agents.

## 5 Conclusion

In summary, we have designed and developed a novel ADC anti‐CD30‐LDM which was prepared by DNA recombination and molecular reconstitution. Firstly, the recombinant fusion protein anti‐CD30‐LDP was prepared, and then, active enediyne AE was integrated into the fusion protein to generate anti‐CD30‐LDM. The novel ADC displays specific affinity and extremely potent cytotoxicity to CD30 overexpressed tumor cells and highly therapeutic efficacy against CD30‐positive tumor model in NOD/SCID mice. The preclinical results presented here, together with the scientific underpinnings, suggest that anti‐CD30‐LDM could be a promising candidate for the treatment of CD30^+^ lymphomas.

## Author contributions

QM conceived and guided the study work. RW performed the experiments, analyzed the available data, and drafted the manuscript. LL performed the HPLC assays. SZ provided support in the animal studies. YL performed the H&E staining and immunohistochemical analysis. XW helped in constructing the expression systems and selecting single clone cells. QM and YZ reviewed the manuscript. All authors read and approved the final manuscript.

## Supporting information


**Fig. S1.** Structures of the antibody‐drug conjugate and its expression vector.
**Fig. S2.** Characterization of antibody‐based fusion protein.
**Fig. S3.** The residual level of free AE after ultrafication analysed by HPLC.
**Fig. S4.** The cytotoxicity of anti‐CD30‐LDP and anti‐CD30‐LDM on Karpas299 and L540 cell lines.
**Fig. S5.** PD‐L1 levels of Karpas299 and L540 cells treated with anti‐CD30‐LDM or anti‐CD30‐LDP.
