# Supplementary material for: A novel enediyne‐integrated antibody–drug conjugate shows promising antitumor efficacy against CD30+ lymphomas
Source: Mol Oncol. 2018 Jan 26;12(3):339–55. doi: 10.1002/1878-0261.12166 (PMC5830626; doi:10.1002/1878-0261.12166)
Supplement: Supplementary file 1 — Fig. S1. Structures of the antibody‐drug conjugate and its expression vector. Fig. S2. Characterization of antibody‐based fusion protein. Fig. S3. The residual level of free AE after ultrafication analysed by HPLC. Fig. S4. The cytotoxicity of anti‐CD30‐LDP and anti‐CD30‐LDM on Karpas299 and L540 cell lines. Fig. S5. PD‐L1 levels of Karpas299 and L540 cells treated with anti‐CD30‐LDM or anti‐CD30‐LDP. [file MOL2-12-339-s001.docx]

**Supplementary Figures**

**Figure S1: Structures of the antibody-drug conjugate and its expression vector.**

**
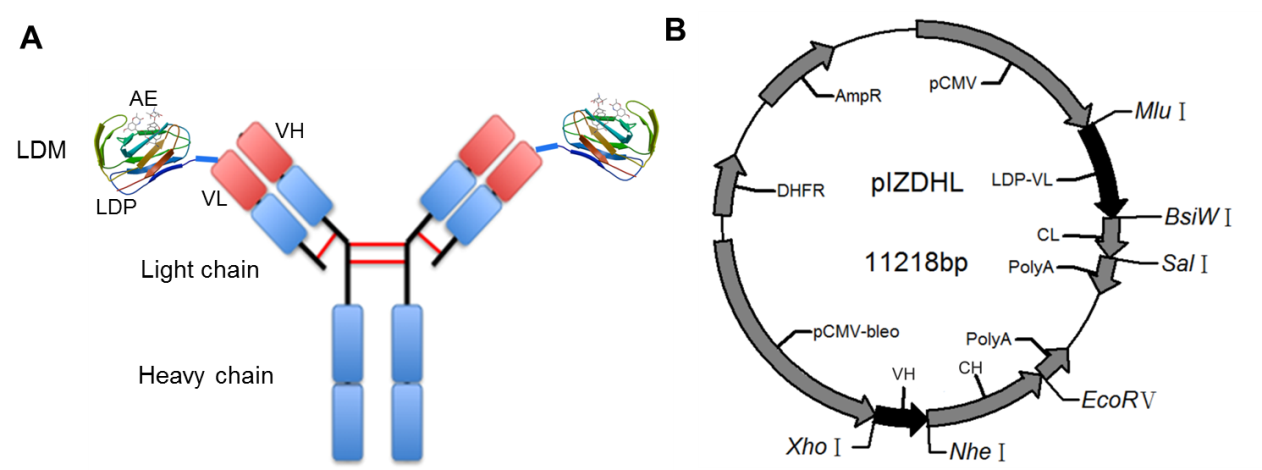
**

**Figure S1:** Structures of the antibody-drug conjugate and its expression vector. **(A)** The schematic diagram of anti-CD30-LDM. **(B)** Schematic view of anti-CD30-LDP expression vector. DNAs encoding the heavy chain and light-LDP chain were cloned into plasmid pIZDHL respectively for expression of fusion protein anti-CD30-LDP in CHO cells.

**Figure S2: Characterization of antibody-based fusion protein.**

**
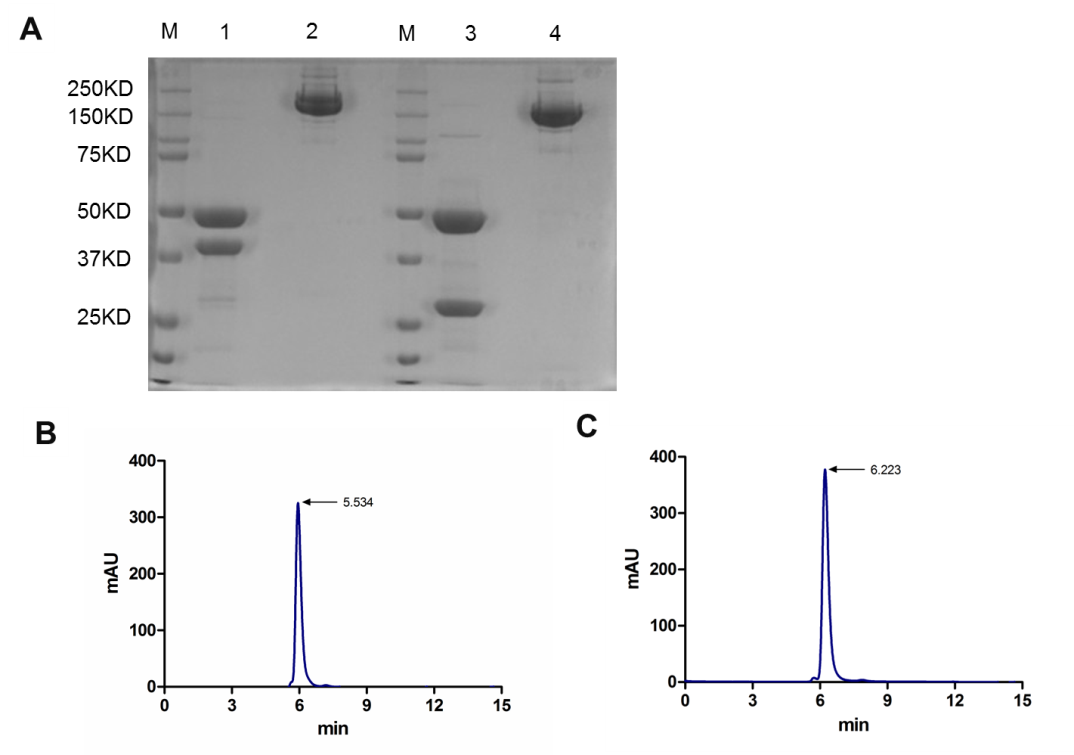
**

**Figure S2: Characterization of antibody-based fusion protein.** **(A)** SDS-PAGE analysis of fusion protein anti-CD30-LDP and parent antibody after purification. Lane M, molecular weight marker; Lane 1, anti-CD30-LDP under reducing conditions; Lane 2, anti-CD30-LDP under non-reducing conditions; Lane 3, anti-CD30 antibody under reducing conditions; Lane 4, anti-CD30 antibody under non-reducing conditions. **(B)** HPLC analysis of anti-CD30-LDP at 280nm. **(C)** HPLC analysis of anti-CD30 antibody at 280nm.

**Figure S3** **The residual level of free AE after ultrafication analysed by HPLC.**

**

**

**Figure S3: The residual level of free AE after ultrafication analysed by HPLC.** After assembly of enediyne-integrated anti-CD30-LDM, the remaining free AE was removed by ultrafiltration and the permeate was detected by reverse-phase HPLC until there was no peak for AE.

**Figure S4 The cytotoxicity of anti-CD30-LDP and anti-CD30-LDM on Karpas299 and L540 cell lines.**

**
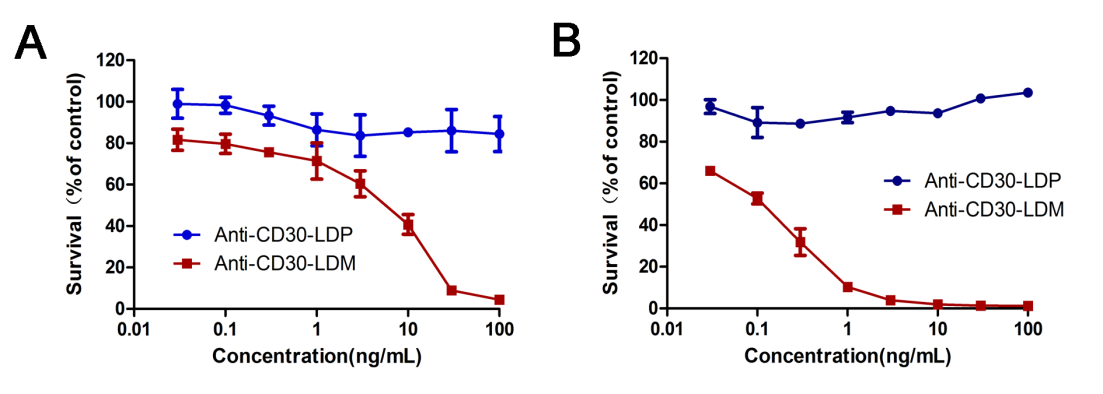
**

**Figure S4: The cytotoxicity of anti-CD30-LDP and anti-CD30-LDM on Karpas299 and L540 cell lines.** Karpas299 **(A)** and L540 **(B)** cells were treated with anti-CD30-LDP and anti-CD30-LDM in a series of concentrations (0.01-100 ng/mL) for 48h. Cell viability was tested by Cell Counting Kit -8 (CCK-8). Results are the mean values ± SD of three replicates.

**Figure S5 PD-L1 levels of Karpas299 and L540 cells treated with anti-CD30-LDM or anti-CD30-LDP.**

**
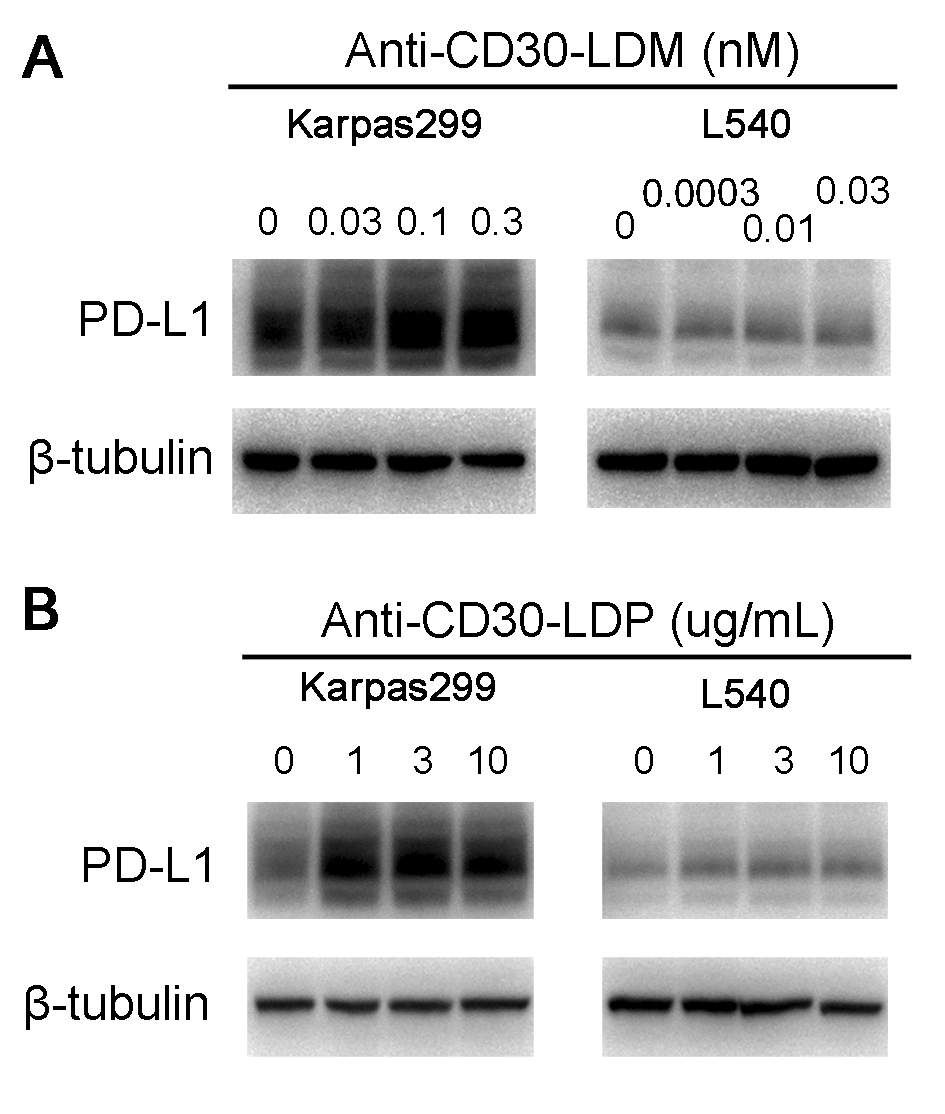
**

**Figure S5: PD-L1 levels of Karpas299 and L540 cells treated with anti-CD30-LDM or anti-CD30-LDP.** Karpas299 and L540 cells were exposed to the appointed concentration of anti-CD30-LDM **(A)** and anti-CD30-LDP **(B)** for 12 hours and samples were collected to analysis the levels of PD-L1. β- tubulin was used as a loading control.
